# “A Soft Death”: Perceptions and Attitudes Toward Palliative Care in Senegal

**DOI:** 10.1093/geroni/igad129

**Published:** 2023-11-17

**Authors:** Hannah I Silverstein, Fursan Sahawneh, Brian D Carpenter

**Affiliations:** Department of Psychological and Brain Sciences, Washington University in St. Louis, St. Louis, Missouri, USA; Department of Psychological and Brain Sciences, Washington University in St. Louis, St. Louis, Missouri, USA; Department of Psychological and Brain Sciences, Washington University in St. Louis, St. Louis, Missouri, USA

**Keywords:** End of life, Francophone, Qualitative descriptive study, Serious illness, Sub-Saharan Africa

## Abstract

**Background and Objectives:**

Palliative care (PC) is a specialty medical service that aims to address the bio-psycho-social–spiritual needs of patients with serious illnesses and their care partners. Despite the value of PC, its expansion in Sub-Saharan Africa has been uneven and particularly underdeveloped in Senegal due to variability in access to training resources and funding. This study sought to understand the current landscape of PC services in Senegal and the barriers and opportunities in its growth.

**Research Design and Methods:**

Semistructured interviews were conducted with six PC clinicians, four caregivers of people with serious illnesses, one driver for a PC service, and one public health professor, all native to Senegal. Questions addressed their experience delivering or receiving PC, perceptions of barriers to implementation and access, and recommendations for additional resources and initiatives. Interviews were translated and transcribed from French into English. Transcripts were qualitatively coded for concepts during open and focused coding to identify themes.

**Results:**

Five themes were identified: (1) the current landscape of PC, (2) barriers to implementing PC, (3) strategies and philosophies in care, (4) unique features of Senegalese culture, and (5) the future of PC.

**Discussion and Implications:**

Our findings demonstrate that PC in Senegal remains an underresourced and underutilized specialty medical service, but work is being done by personally committed clinicians. Results can inform PC expansion by highlighting important cultural factors influencing care in Senegal, as well as the need to expand training opportunities for clinicians, increase education of other medical providers and the public about the nature of PC, integrate PC into the healthcare system, and expand research to evaluate the impact of these resources. PC has the potential to be an important force for improving the quality of life for Senegalese patients and their care partners.

**Translational Significance:** Palliative care remains an underresourced medical specialty in Senegal. This study synthesized perspectives from key informants to identify critical needs to promote expanded access to palliative care. This information could guide governmental and nongovernmental organizations in expanding education of providers, educating the public about palliative care, growing inpatient and outpatient services, implementing creative models and locations of service delivery, and promoting research to document the impact of palliative care. Findings are also relevant to healthcare and regulatory policies that influence the provision of palliative care. These efforts should be undertaken with sensitivity to the historical and cultural context in Senegal.

The World Health Organization (WHO) defines palliative care as improving the quality of life of patients, families, and their caregivers who face challenges related to a life-threatening illness, whether physical, psychological, social, or spiritual (WHO, 2020). Despite the growing global need for palliative care services, only about 14% of people who need palliative care receive it; of those who do not, 78% live in low- and middle-income countries (WHO, 2020). Palliative care has expanded in these low- and middle-income countries (Abu-Odah et al., 2020; Hannon et al., 2016), both within and outside Africa, but challenges remain. For example, opinions about what information to share with patients about serious illness and what constitutes a “good death” vary widely across places and cultures (e.g., from different regions, see these reports from Taiwan (Lin et al., 2019), Botswana (Efstathiou et al., 2016), and Latin America and China (Basu et al., 2013)). Likewise, palliative care remains underresourced in several regions, such as the continent of Africa (Agom et al., 2021), the Eastern Mediterranean (Fadhil et al., 2017), and the Middle East (Zeinah et al., 2013).

Considering this need and these challenges, palliative care services in Africa have expanded thanks to policy developments such as the World Health Assembly’s 2014 resolution to strengthen palliative care throughout the life course (Centeno & Rhee, 2018) and the WHO’s public health strategy for palliative care (Stjernswärd et al., 2007), with its focus on developing and integrating palliative care services, policies, medicines, and education on a national level. Although palliative care is provided at all service levels, it is often provided in-home, as it is seen as more feasible and more acceptable by patients (Hannon et al., 2016), and thus in-home services have expanded throughout Africa. Recent dissemination of palliative care can be credited to local providers, such as the community volunteers who help support Hospice Africa Uganda activities (Loth et al., 2020). Yet, estimates suggest that only 5% of people in Africa in need of palliative care are able to receive it (Downing et al., 2015). Furthermore, past research on palliative care in Africa has often focused on countries with relatively well-developed programs such as Uganda, Kenya, and South Africa (Downing et al., 2015; Drenth et al., 2017; Fraser et al., 2017; Grant et al., 2017), with less of a focus on West African, Francophone countries such as Senegal, where palliative care is less-developed (Afolabi et al., 2021; Hamdi et al., 2017; Rhee, Garralda, Namisango, Luyirika, de Lima, Powell, Robinson, et al., 2018). Furthermore, scholars have noted gaps in research on palliative care in Sub-Saharan Africa (Afolabi et al., 2021; Fraser et al., 2017; Hamdi et al., 2017; Harding & Higginson, 2005; Harding et al., 2013). In fact, Fraser and colleagues (2017) concluded in their review of palliative care in Uganda and Kenya that research is the most neglected area for informing clinical interventions, and a recent synthesis of current research evidence and gaps highlighted many information needs, most acute in northern and West Africa (African Palliative Care Association [APCA], n.d.a).

## The Context of Palliative Care in Senegal

As in other locations around the world, within the Senegalese medical model, palliative care seeks to alleviate pain and improve the quality of life for individuals with serious, life-limiting illnesses and their families. These illnesses can be terminal, such is the case with cancer, or chronic, such as diabetes and hypertension. Senegal has remained a country with “isolated palliative care provision” (Clark et al., 2020, p. 801). Based on the WHO public health strategy for the implementation of palliative care, Senegal ranked 28th among Sub-Saharan African countries (Rhee, Garralda, Namisango, Luyirika, de Lima, Powell, López-Fidalgo, et al., 2018). Similarly, according to the APCA Atlas of Palliative Care in Africa, last updated in 2017 (Rhee, Luyirika, et al., 2017), in Senegal there were only 0.06 palliative care physicians per 1,000 people. The country had only five formal organizations providing palliative care, with just four of the 39 hospitals in Senegal providing inpatient palliative care and one home-based palliative care service (Rhee, Luyirika, et al., 2017).

It should be noted, however, that in recent years, the government in Senegal has made several important efforts to bolster palliative care structures. For example, in 2016, the Ministry of Health and Social Action changed regulations so that outpatients could receive morphine for a maximum of 28 days, an increase from the previous 7 days. Likewise, the agency responsible for procuring medicines, the Pharmacie Nationale d’Approvisionnement, has been expanding its efforts, increasing the national morphine supply to align with needs (International Narcotics Control Board, 2023b).

Cultural context plays an important role in delivering effective palliative care services in Africa, and that is true in Senegal as well (Afolabi et al., 2021; Harding & Higginson, 2005). For example, traditional healers play an important role in providing care (Agom et al., 2021). Additionally, stigma around certain terminal illnesses such as HIV/AIDS in some African countries has been reported in the literature (Gysels et al., 2011), as well as a hesitancy to talk about death as this is seen as taboo in parts of Africa (Ekore & Lanre-Abass, 2016). Beyond healthcare, religion also plays an important role in Senegal’s cultural context, given that 95.9% of the Senegalese population is Muslim, and 4.1% is Christian (U.S. Department of State, 2020). Past research has identified that religious beliefs in Africa influence the implementation of palliative care, such that religious patients will place their faith in God to cure them and not seek medical treatment (Agom et al., 2021). Fearon et al. (2019) further explain that in Islam, it is believed that there is a cure for every illness, whether it is available or not yet discovered, and thus this belief may influence medical treatments to favor prolonging life. Likewise, underlying socioeconomic and cultural dynamics are important when delivering palliative care in Africa (Afolabi et al., 2021). At the same time, given the diversity of cultures present in Africa, it is important to understand the specific cultural context of Senegal on its own, rather than generalizing from other countries in Africa. Regarding the history of Western medicine in the country, Governor General Ernest Roume first introduced Western medicine to Senegal at the beginning of the twentieth century through public health policy to control the spread of infectious diseases (Bignon, 2012). It is important to note that this expansion coincided with French colonialism of West Africa, resulting in the displacement of indigenous Senegalese people, economic exploitation, and efforts to undermine traditional African healing practices (Tilly, 2016). Healthcare institutions were largely concentrated in cities, and in 1982, the United States Agency for International Development (USAID) helped implement an initiative to bring healthcare systems to more rural communities (USAID, 2017). Despite this, there is a concentration of healthcare services offered in Senegal’s capital city, Dakar, with a lack of healthcare services available in more rural parts of Senegal (Banyan Global, 2015).

This study sought to answer calls for research on palliative care in Sub-Saharan Africa and fill gaps in the literature by investigating the attitudes and perceptions of palliative care specialists and consumers in Senegal to inform future palliative care expansion and implementation. Furthermore, the present study seeks to illuminate realized and potential interactions between palliative care providers and public health officials that are crucial for the provision of palliative care in Senegal.

## Method

### Participants

Our goal was to recruit physicians and laypeople who had provided care to someone with a serious illness in and around the city of Dakar. The research team recruited both clinicians and informal caregivers to document perspectives from a range of individuals who provide care to those with life-limiting illnesses. The research team was particularly interested in the perspective of informal caregivers, given their direct caregiving experience and as observers of formal care, in contrast to the perspectives represented in past studies, mainly palliative care clinicians and experts delivering care (e.g., Clark et al., 2020; Powell et al., 2014; Rhee, Garralda, Namisango, Luyirika, de Lima, Powell, Robinson, et al., 2018; Tadipatri et al., 2021). Potential physician participants were identified by searching for palliative care physicians in Dakar online by using the search terms “palliative care in Senegal,” as well as from referrals by other physicians. Clinicians worked at either a hospital, a private clinic, or a free in-home palliative care service. Potential informal caregivers were identified by staff at one organization that provided palliative care services and by suggestions from program administrators. One participant was a professor of public health history in Senegal who was asked to participate after giving a lecture. Potential participants were contacted via email, telephone, or text with the assistance of a local administrator who could communicate in the local dialect, Wolof. We approached 14 potential participants, with one clinician unavailable due to time and another who did not respond.

Between May 2022 and July 2022, we recruited 12 participants, all Senegalese natives who currently worked and/or lived in and around Dakar. As such, all participants lived and/or worked in urban settings. Participants ranged in age from 21 to 65 years (*M* = 42, *SD* = 17.59) and were evenly divided between male and female. Six of the participants were clinicians who either worked for a free in-home service, a private clinic, or a public hospital (see Table 1). One participant was a driver who worked for a free in-home service (participant 3). Four of the participants were informal caregivers of patients with long-term illnesses (participant 7) or terminal illnesses (participants 10, 11, and 12; see Table 1). Two participants were caregivers who had received palliative care services (participants 11 and 12). Finally, one participant was a public health professor with historical knowledge of medicine in Senegal within an African cultural context (participant 5).

**Table 1.** Participant Characteristics

Participant	Gender	Professional Discipline	Years in Profession	Location of Services	Palliative Care Specialist/Training	Religious Affiliation
1	M	Nurse	2	In-home	Yes	Unknown
2	F	Nurse	~1	In-home	Yes	Unknown
3	M	Driver for Palliative Care Service	1	In-home	No	Unknown
4	M	Physician	10	Private clinic	Yes	Unknown
5	M	Public Health Professor	N/A	N/A	No	Unknown
6	F	Gynecologist; President of Palliative Care Service	2	In-home	No	Unknown
7	F	Caregiver	5	N/A	No	Muslim
8	M	Physician	2	Private clinic	Yes	Unknown
9	F	Physician	8	Public Hospital	Yes	Unknown
10	F	Caregiver	Unknown	N/A	No	Muslim
11	M	Caregiver	2	N/A	No	Catholic
12	F	Caregiver	2	N/A	No	Catholic

### Procedure

This study was approved by the Institutional Review Board at Washington University in St. Louis. We used a qualitative descriptive approach (Kim et al., 2017) to elicit participants’ perspectives on palliative care provision. A semistructured interview guide was developed by the investigators based on a review of the literature on the development of palliative care in Africa (Connor et al., 2020; Fearon et al., 2019; Hamdi et al., 2017; Rhee, Luyirika, et al., 2017) and similar studies conducted in other parts of the world (Midlöv & Lindberg, 2020; Traister et al., 2018; see Supplementary Material Sections A, B, and C). The interview guide was slightly modified depending on the participant and their methods of delivering or receiving care (e.g., in the home or a clinic).

All interviews were conducted by the same research team member (H. Silverstein) during June 2022 and July 2022 in Dakar, Senegal. After recruitment, the interviewer met 11 of the participants to conduct their semistructured interviews in person, at their place of work or their homes. Before beginning the interview, participants consented to participate in a study on the “experience of palliative care in Senegal from the perspective of palliative care providers and caregivers of patients receiving palliative care.” One interview was conducted over the telephone at the participant’s request. Ten interviews were audio recorded using a smartphone. Two participants declined to be recorded, and the interviewer took detailed notes during the interview. A translator was present to help translate between French and Wolof into English for five interviews. Interview length varied from 15 to 30 min. To thank participants, each received a small gift as is customary in Senegalese culture.

### Data Analysis

Two translators, an upper-level undergraduate studying French and a doctoral student in French, translated the 10 recorded interviews into transcripts from French into English. To ensure reliability, each translated one transcript, then reviewed the work of the other translator and met to resolve discrepancies. Once translation standards had been agreed upon, the remaining transcripts were then evenly divided among the two translators to translate individually. The translators nominated especially difficult passages to be reviewed by the other translator if necessary. A professor at the first author’s institution who is a Senegal native was consulted to assist with translating Wolof into English, which was present in varying degrees in seven of the interviews. A member of the research team (H. Silverstein) then reviewed the transcripts and redacted any personal information before qualitative coding began.

The research team (H. Silverstein, F. Sahawneh, and B. D. Carpenter) met to review two transcripts and inductively develop an initial coding scheme using open coding (Saldaña, 2021). Next, each team member returned to the initial two transcripts and repeated coding with the coding scheme. Adjustments were made to several codes to clarify their operationalization and scope and finalize the codebook (see Table 2). Transcripts were then uploaded to Dedoose, a qualitative coding software (Dedoose, 2023). Two research team members (H. Silverstein and F. Sahawneh) each coded all nine remaining transcripts using focused coding (Saldaña, 2021), coding two to three transcripts at a time and meeting weekly to discuss and resolve discrepancies. During these discrepancy discussions, coders would clarify code definitions in the codebook and add or delete codes. This iterative process helped coders identify emerging themes by comparing codes and establishing emerging patterns while using concept coding throughout (Saldaña, 2021). A research team member (H. Silverstein) then synthesized the codes into key themes (see Table 2) based on a clustering of the most prevalent codes in the transcripts.

**Table 2.** Qualitative Codes

Code Name and Subcodes	Code Definition	Code Application	
		Example	Code(s) Applied
Barriers to palliative care implementation -Death as taboo/stigma around death -Issues with care coordination -Lack of access to resources -Lack of awareness among the public -Lack of funding -Lack of transportation	This applies when participants mention concrete or abstract barriers/obstacles to implementing palliative care in Senegal; specifically says X is a problem, that is, participant won’t talk about illness, so they won’t take chemotherapy.	“Well, the difficulties … uh. I can say that it’s uh, morphine access. Medication. Because it’s hard to find someone who is in pain and you can’t do anything to help … The difficulty is really with respect to morphine access.”—Participant 1	**Lack of access to resources**
Care and services provided by caregivers -Custodial care -Emotional/psychological support -Medical symptom management -Spiritual support	This applies when a caregiver describes the care they themselves provide to patients or other caregivers who they work with, that is, other family members.	“Assisting! Prayer! We pray a lot. Since we are Muslims, we pray a lot for the sick and we also take good care of them, especially that! Anything they want from us, we do it!”—Participant 10	**Spiritual support** and Strategies and philosophies in providing care
Care and services provided by clinicians -Custodial care -Emotion/psychological support -Family support/education -Location of care -Medical symptom management -Spiritual support	This applies when a clinician mentions the concrete, tangible care they provide to patients. This can also include when caregivers mention the care that clinicians provide.	“Here we can do psychological work … we can do coaching, for patients and for family, we can do acupuncture.”—Participant 6	**Care and services provided by clinicians, Emotional/psychological work and Family support/education**
Evolution and current landscape -Different patient groups -Gaps in care driving creation: Economic hardship/poverty and lack of accommodations -High demand -Novelty	This code applies whenever a participant discussed how the field of palliative care has developed over time, as well as describing what the conditions of palliative care look like currently in Senegal.	“My brother-in-law had high blood pressure.” —Participant 10	**Different patient groups** and Training and work history
Features of Senegalese culture -Comparing Senegal to other cultures -Death as taboo/stigma around death	This applies when participants mention unique facets of Senegalese culture to describe the general population (doesn’t need to be related to palliative care necessarily), that is, mentioning traditional medicine.	“Well! In the African mindset, illness is something secret. One should not let others know that we are sick. One should not give that much information about one’s illness.”—Participant 5	Disclosing information and **Features of Senegalese culture**
Future of palliative care in Senegal -Expanding locations -Increased awareness and educating patients and families -Increased funding and government involvement -More training for clinicians	This applies when participants describe what they would like/think the future of palliative care looks like in Senegal (usually in response to the question of what could be improved). This also includes what strategies participants will use in the future to overcome barriers to care.	“First make it [palliative care] known, make it known to the population. That it exists. People need to know that it exists, first of all. [laughs] That it exists, first of all.”—Participant 8	**Increasing awareness and educating patients and families**
Palliative care as interdisciplinary	This applies when participants mention the field of palliative care being interdisciplinary and/or involving specialists from other field, that is, working with acupuncturists or cardiologists.	“So recently a physician who is in … at [X] hospital, and who put us in touch with a patient with end-stage stomach cancer who needed palliative care. We started taking care of that patient.”—Participant 2	Different patient groups and **Palliative care as interdisciplinary**
Participant characteristics -Personal feelings/attitudes toward care -Reasons for entering palliative care: Passion for caring for older adults/sick people, Personal experience and recognizing larger societal issues -Training and work history: Duration in the field, Time in organization & Training	This applies to information that will be used in Table 1, as well as participants’ personal beliefs toward the field of palliative care.	“Because I had, uhm, personal experience, a member of my family had cancer and I had … I didn’t have the possibility of having palliative care for him.”—Participant 6	**Personal experience**
Strategies and philosophies in providing care -Accompaniment -Disclosing information -Influence of religion -Relationships with family and patient -Relieving suffering	This code applies whenever a participant mentions their present strategies or philosophies in providing care; could be describing how they provide care or how others in general provide care; more abstract and different from merely stating the concrete, physical services they provide.	“You need to have someone who has a sweet heart who can support him [patient], someone who can bear a lot to deal with him.”—Participant 7	**Strategies and philosophies in providing care**

*Note*: The text in bold in the “Code(s) Applied” column indicates the application of the code being defined. Other codes listed here would also be applied and demonstrate the ways in which codes overlap.

## Results

Several unique themes emerged from the interviews regarding perceptions and attitudes toward palliative care and terminal illness in Senegal. In general, themes in conversations with clinicians overlapped with those in conversations with caregivers, so perspectives from both types of participants are reflected in the narratives below. First, participants discussed the current landscape of palliative care in Senegal, noting its infancy as a field, their reasons for joining, and the types of patients they serve. Next, participants outlined their view of current barriers to implementing palliative care in Senegal, including educational and structural impediments. Third, participants described their strategies and philosophies when providing serious illness care. They discussed unique features of Senegalese culture that influenced their practice. And finally, participants described their hopes for the future of palliative care in Senegal.

### Current Landscape of Palliative Care

Participants, especially clinicians, frequently described the novelty of palliative care in Senegal, even in the face of high demand for palliative care services. In a comment echoed by others, one clinician said, “Recently we have been receiving calls from everywhere, including some physicians who know about our association and call us to request our services because they know what we have been able to do in just two years … It’s remarkable!” (P1). Many of these clinicians had introduced palliative care to their practice even though palliative care is not integrated into Senegal’s national healthcare system. For example, one clinician described bringing palliative care to her hospital in 2014 and now helps train medical students in palliative care (P9). She continued to explain that there is no formal training in palliative care in Senegal, thus she received her training abroad in Uganda and the United States. Several participants worked at an organization formed in 2020 that provides free, in-home palliative care services. They mentioned the immediate and high demand for services as soon as the organization started, an indication of the high need in the community: “The demand is so big. In every house there is maybe someone that needs palliative care” (P6). As there is no nationally funded palliative care program in Senegal, this organization, as well as the private clinics, receives a substantial number of patients, both within and outside Dakar.

Most of the participants were new to the field of palliative care. Many clinicians had been working in the field of palliative care for only a couple of years (P1, P2, P3, P6, and P8) and only two clinicians interviewed had been in the field for close to a decade (P4 and P9). Clinicians described receiving special training in palliative care that was outsourced from other countries: “I was able to go to Uganda for training. Yes, for palliative care training. I was there for 3 weeks and did online classes for 3 months. Yea. So, I have the certificate in palliative care nursing.” (P1). Caregiver experiences in caring for those with long-term needs ranged from 2 to 5 years. When asked why they were interested in providing palliative care services, clinicians and caregivers reported three main motivators. First, they said they felt a passion for caring for older adults: “I like to help the sick, I like to take care of the sick. As long as I have the strength, I will keep doing it” (P10). Second, personal experience was an important factor for some participants. One clinician (P6), the founder of an organization that provided in-home palliative care service at no charge, explained that her family member had cancer, and she wished there had been palliative care services available. Third, some clinicians also highlighted larger structural deficits in the healthcare system that they wanted to address. For example, a primary care physician (P8) noted that many patients do not have the means to get to the hospital: “So I thought, why not create something or get involved in an organization who go near the sick. They don’t have the possibility to go to the hospital.”

In describing their work, clinicians also mentioned the interdisciplinary nature of their palliative care services. Clinicians reported calling in specialists, depending on the case, ranging from endocrinologists, cardiologists, and providers more skilled in prescribing opiates. Said one palliative care nurse (P1), “And we also have particular cases where it surpasses our expertise, we call them, or even if, for example, we need access to morphine … we call our doctor who can prescribe the ordinance.”

Participants also described the wide range of clinical issues they addressed when providing palliative care services. These included unmanaged hypertension, untreated diabetes, cancer, and poststroke care. Participant 10, a caregiver, described the health conditions of her relatives that she cared for: “My brother-in-law had high blood pressure … my mother-in-law had cancer.” Participant 8, a primary care physician with training in palliative care, described caring for “… the bedridden and those suffering from strokes.” Participants working at an outpatient nonprofit palliative care service also described caring for patients of all ages, from various economic backgrounds,

It’s people from all categories. Be it the poor, the wealthy, everyone! In the beginning, we started with people in need, those who did not have money. We came to realize that even people with financial stability also get sick, are not happy, and live with anxiety. So, we give them relief and help them to move through the stages of illness … We accept everyone. (P2)

### Barriers to Implementing Palliative Care

Clinicians cited several difficulties in implementing palliative care in Senegal. As noted by several clinicians, morphine availability is a perennial problem in Senegal, compounded by other impediments to prescribing: “The quantity of morphine available to Senegal is low. You can’t go to buy it at the pharmacy, so we go to the hospital, and when we can’t buy it at the hospital, it’s complicated.” (P1). Nurses cannot prescribe opiates, so they may have to call physicians to prescribe morphine, and private associations must work with hospitals to get access to morphine. Additionally, caregivers expressed the difficulty of seeing their loved ones in pain: “It was difficult for her [patient] and I wasn’t able to look at her because I saw her suffering.” (P12). In addition to coordinating morphine access, private associations have difficulties coordinating patients with hospitals. Clinicians working at private associations explained that there is no formal referral system in place and that they must rely on hospital staff to refer patients one by one: “Today, for example, we still have difficulties working with big hospitals … So we have no agreement with hospitals. It is not easy for us to get patients from those hospitals” (P1).

In addition to these structural barriers, participants described difficulties with transportation and funding as limiting the implementation of palliative care. Many patients struggle to transport themselves to the hospital, hence the development of in-home services: “Sometimes there are parents that are sick, relatives and it’s that they can’t move themselves to go to the hospital to get care” (P8). A lack of funding for palliative care services, in addition to the high cost of health care and economic hardship experienced by some patients, further exacerbates these structural barriers: “People are so poor that sometimes they can’t even buy … dressings/bandages” (P6); “There is a lack of means as well. There is a lack of money” (P8). One clinician (P1) further elaborated that there is a lack of funding to train more palliative care workers since the demand is so high:

So we need for the state to put more means into it so that we can get more workers … what I’m telling you is that people are in their houses, they are suffering … treatment has become really difficult. And the hospital, you go to the hospital and there are so little people that can take care of you. So, people stay at their homes and suffer. So, this, we need for the state to help.

Clinicians also explained how public perceptions and attitudes around palliative care and the end of life prevent people from benefitting from their services. Some participants explained that there is simply a lack of understanding of palliative care among the public and that, “Many do not know what palliative care is” (P2). Others explained that people have heard of palliative care, but they misunderstand what it is or are averse to its association with death and dying: “The population is scared of it, they don’t know palliative care” (P1). Clinicians providing free in-home services explained a unique challenge in that people are suspicious about using their service precisely because there is no charge: “But the other difficulty is that we do care for free. Everything is free, which is suspicious … And a lot of people, when it’s expensive, it’s good. When it’s free, there’s a problem” (P6).

In another comment related to public education, one participant mentioned traditional medicine as a barrier, because people often go to traditional healers such as a marabout, who is seen as a religious leader in Islam as practiced in Western Africa (Gemmeke, 2009), for medical help as opposed to the hospital or a clinic: “That’s to say, they [the public] don’t know that there are maybe doctors that exist. Some know that they are just marabouts who will prescribe weird things like showering. They think the use of traditional medications will solve their problem” (P8). However, this sentiment was not expressed by other participants.

### Strategies and Philosophies in Care

Caregivers and clinicians were similar in their descriptions of how they provided care and the strategies they used to overcome barriers to implementing care.

When asked about how much information is shared with patients or family members regarding their illness, participants gave varying responses. One participant who is a professor of public health history in Senegal explained that there is a cultural norm of medical secrecy around illness in Senegal and that only the patient and close relatives are typically informed about the illness: “In the African mindset, illness is something secret. One should not let others know that we are sick. One should not give that much information about one’s illness” (P5). This reticence even applies to prognostic information in comparison to Western cultures, as one palliative nurse described: “Maybe I can talk to a family member, give him advice, and ask him to be closer to the patient, but not tell him exactly what is going on because realities here are different from those of the United States” (P2). Caregivers also elaborated that they will not tell the patient they are going to die, but instead continue to hope for their recovery: “Because we do not explain exactly whether the patient will die at such a date. We don’t say anything. So, we still keep faith that the patient will recover. So, we keep the faith, and we think one day he/she will recover, he will be healed, you know!” (P10). Some clinicians explained that they try to accommodate patient perceptions when disclosing information to overcome stigma, such that they tell the patient what they need to know, but not that they will die so as to scare them away from treatment. As one physician said,

So when we arrive, we aren’t going to dig around a lot about our knowledge. We produce just what the people know. We can’t give and give them information that they don’t know. So, we wait and see if people ask questions, and we respond. But us, we’re not going to you know, we respond to questions, and we give information just up until what the person knows. (P6)

In contrast, other participants explained that they tell patients and families everything to keep the family informed and get truly informed consent from the patient:

Every test that we do. We tell them we are doing this test. And the tests are to look for those things. Once we have the results, we explain so they know. For every analysis, we tell them the purpose, and when we get the results, we tell them what it revealed, and for treatment, we will do this or that. Anyways, there is no secret between us, the sick person, and their family. (P8)

When defining what palliative care is and the services it provides, participants emphasized a holistic treatment that accompanies a person along their illness trajectory. Clinicians at the organization providing free in-home services repeatedly used the term “accompaniment” to describe their philosophy in providing palliative care:

It’s almost the definition of palliative care—accompanying people with chronic illnesses. We will care for them so that we can save their leg so that we can accompany someone who is injured, who is paralyzed, we are going to help them and care for them, we will do physiotherapy also. If you have cancer and are in pain, there you have it, it’s accompaniment. (P6)

Participants defined palliative care as a global approach to the treatment of illness, incorporating not only management of medical symptoms but also providing custodial care in addition to offering emotional and spiritual support: “Palliative care is holistic. More specifically, it’s psychological, social, physical, and spiritual” (P1). Pain management is an important focus: “We give painkillers to improve quality of life, so he has a soft death … So he has a short death, meaning to die with no difficulty, with less pain” (P8), but so, too, is addressing other forms of suffering: “We are here … so that they don’t suffer, so that they aren’t in pain so that they find the comfort, you know? It’s the first things that we do” (P1).

Several participants also mentioned the close relationship that develops among palliative care specialists, patients, and family members that influences how palliative care is delivered. Clinicians said they develop strong relationships with patients and family members and become a part of the family. One physician noted,

It’s no longer a relationship between a doctor and his patient. It’s now doctor and family. There are people we have known for 5, 6, 7 years … At some point, it becomes a family. So no longer a relationship between doctor and patient, but now it’s doctor and family. We become a member of the family. (P8)

A caregiver also brought up their close relationship and ability to rely on their clinicians, which helped with grieving: “For us, the relationship that we had with, for example, the doctors and the patient, it really helped us, they really, really participated in the healing process.” (P11).

### Unique Features of Senegalese Culture

Among features of Senegalese culture that participants said influenced palliative care, religion was described as the most prominent. All four of the caregivers mentioned their religious affiliation (Islamic or Catholic), and described using prayer in their care, “Prayer! We do nothing but pray. We pray for the patient, we pray, we pray” (P10), and explained how their faith motivates them to care for others, “She [the caregiver] says she is doing that … in God’s sake, she’s doing that for free. She is not doing that to be paid, she’s not doing that … you know for any purposes. She’s just doing that because she’s willing to do it” (P7). Others described the importance of God in explaining illness and death to patients: “Because if someone is sick and afraid of dying, you tell him/her, ‘No! God is in control, you will not die, take your medicine. Physicians can say you will die but God is there, and you can live for years to come, okay?’” (P10). Clinicians also elaborated on this, explaining how they incorporate religion into their care. For example, when patients consult religious leaders such as Imams, clinicians will collaborate with these leaders to care for the patient. As one palliative care nurse expressed, “Sometimes also we have a patient that trusts the Imam of the area. Sometimes we need to speak with the Imam because it was only the Imam who could make him understand, you know?” (P1). And prayer and other traditions can be brought into care: “Sometimes, when patients would get to the terminal stage … there is a Quran verse … that is recited before the patient to ease their pain and escort them to the afterlife” (P5).

Participants also mentioned secrecy and stigma around end-of-life issues. One physician expressed that in Senegal “death is taboo. We don’t want to talk about it” (P6), whereas a caregiver explained that they do not use the word “terminal stage.” Participants reported that families will sometimes hide their loved one’s illness from outsiders, as well as from the patient themselves who is exhausted from fighting their disease. When asked if treatment for the patient is ever stopped, caregivers 10, 11, and 12 said that treatment was never stopped. Finally, a public health professor (P5) pointed out that end-of-life wishes are not documented in Senegal in the same way they are in the West, which presents difficulties related to decision-making and treatment cessation, particularly considering a commonly held belief that only God decides when you die, not a physician.

### Future of Palliative Care

Clinicians expressed several aspirations regarding the future development of palliative care in Senegal. The president of the organization that provides free in-home services repeatedly expressed a desire to expand their services, hoping to establish outpatient clinics in every region of Senegal: “Why not have a[n out-patient palliative care] house like this where each person can live, in each region” (P6). Clinicians (P1 and P8) acknowledged, however, that greater assistance from the government is needed to expand services: “Put the necessary means in place. By that, I mean ambulances, cars to take staff members to patients’ homes. All this should be subsidized by the government. Because home consultation is a little bit expensive. It’s the means of transportation, the consultation subsidy” (P8). One clinician (P9) expressed a wish for more involvement and funding from the government and specifically the minister of health to integrate palliative care into the national healthcare system and into the training of physicians.

Finally, several clinicians mentioned the importance of efforts to increase awareness of palliative care in the public to improve its use and development. This can occur with individual families in which someone is ill, as explained by one palliative care nurse: “At home, we should inform, and raise awareness of the family in that sense. Inform them and raise awareness about palliative care … because many do not know what palliative care is” (P2). Likewise, because people cannot use palliative care services if they are not aware of them, clinicians expressed that increasing awareness could help reach more people who are suffering, as noted by one physician who said, “make it [palliative care] known to the population that it exists in Senegal and exists well in Senegal, even if it’s not as big of a structure, but it exists and we get by with the means at hand” (P8).

## Discussion

This qualitative interview study contributes to a growing body of literature on palliative care development in Sub-Saharan Africa by focusing on palliative care in Senegal from the perspective of clinicians and informal caregivers for people with serious illnesses. Our findings document the current state of palliative care in Senegal, barriers to its implementation reflecting results from other African countries reported by Rhee, Garralda, Namisango, Luyirika, de Lima, Powell, Robinson, et al. (2018), goals for its future development that support the theorizing of Stjernswärd and colleagues (2007) based on the WHO’s public health strategy for expanding palliative care, as well as important features of Senegalese culture influencing care.

Past research on the development of palliative care in Sub-Saharan Africa has primarily documented its expansion in countries with more extensive programs, such as Uganda, Kenya, and South Africa (Downing et al., 2015; Drenth et al., 2017; Fraser et al., 2017; Ntizimira, et al., 2014). Less common are reports on the state of palliative care in Francophone countries in West Africa (Human Rights Watch, 2015). In Senegal, there have been recent and rapid changes not previously documented in the literature. Indeed, one of the organizations represented in the current study did not exist at the time of previous research (Clark et al., 2020; Hamdi et al., 2017; Rhee, Garralda, et al., 2017; van der Plas et al., 2020) and emerged to fill a gap in the care continuum. Despite these developments, overall, palliative care services in Senegal appear to be inadequate to meet current demands, based on the number of patients who could benefit from symptom management, care coordination, and other supportive services (Hamdi et al., 2017). In addition, palliative care services are relatively disjointed and uncoordinated and often only available because of the singular efforts of organizations (and sometimes, heroic individuals). Unlike those of some other African nations, the Senegalese government has yet to develop a plan to integrate palliative care into its national public health system (Gueye, 2016), which could help support the efforts of these organizations and individuals. Despite modest support and coordination by the state, a few vibrant organizations and dedicated individuals are working to deliver and promote palliative care.

Clinicians in this study also highlighted specific practical barriers to implementing palliative care, some of which have been previously documented but nonetheless persist. Many clinicians cited difficulties with opioid access, as morphine is managed by the state and hospitals, and specialists may be required to prescribe it. Caregivers offered corroborating accounts, mentioning difficulties seeing their loved ones in pain without the benefit of routine access to opiates. The Senegalese government has taken important steps to increase access to morphine in public hospitals and train clinicians on prescribing (Human Rights Watch, 2017); however, these efforts remain concentrated in Dakar and do not address difficulties in prescribing morphine to patients being treated by private organizations. According to the International Narcotics Control Board, a United Nations agency, consumption of opioids for pain management in Senegal, as in other Western and Central African countries, is less than one defined daily dose per million inhabitants per day, far below what is needed for adequate pain control in the population (International Narcotics Control Board, 2023a). These issues with access are not unique to Senegal and have been documented in other African countries (Centeno & Rhee, 2018; Grant et al., 2017; Ntizimira et al., 2014). However, some countries, such as Uganda, have been able to overcome these barriers by passing legislation to allow nurses to prescribe and train clinicians on morphine use to avoid underprescribing opiates related to opiophobia (Jagwe & Merriman, 2007; Rhee, Garralda, Namisango, Luyirika, de Lima, Powell, Robinson, et al., 2018). Clinicians also identified challenges in coordinating care with hospitals. Dakar lacks a structured referral system that would connect hospitals to private organizations providing palliative care. Past research has identified solutions to overcome this barrier and improve partnerships between programs to identify patients with palliative care needs sooner (Downing et al., 2015; Grant et al., 2017; Nwogu et al., 2016).

From a public health perspective, both clinicians and caregivers noted that a lack of knowledge about palliative care represents a major barrier to uptake. Quite simply, some clinicians do not know about the benefits of palliative care, which limits referrals, and members of the public (patients and caregivers alike) also do not know what palliative care is and how it can help, resulting in a delay of referrals. To overcome public misunderstandings, countries like Uganda have undertaken initiatives to educate the public and encourage acceptance by using the media and community volunteers to advocate for palliative care (Drenth et al., 2017; Fraser et al., 2017). Our participants also identified funding as a structural barrier and called on the Senegalese government and its Ministry of Health to make palliative care a priority, in line with the World Health Assembly’s 2014 resolution to strengthen palliative care (Centeno & Rhee, 2018). Given its relative novelty in Senegal, clinicians expressed a desire for more funding, which has similarly been expressed by palliative care clinicians across Africa (Loth et al., 2020). Countries with more developed palliative care systems, such as Kenya and Uganda, can serve as useful models (Fraser et al., 2017). In these countries and others with successful palliative care programs, funding came because of advocacy on a national level by organizations such as the Kenya Hospice and Palliative Care Association and the Hospice and Palliative Care Association of South Africa to invest in palliative care and integrate it into their health system. One contribution of the current study has been to identify that while there have been calls for more coordinated efforts to implement palliative care across the continent from the African Palliative Care Association (APCA, n.d.b.), implementation has been uneven, with success in some countries, but lagging implementation in others. This has led to disparities in access to palliative care *within* the continent that need to be addressed. Strong advocacy for equity is paramount, even while acknowledging different levels of resources across countries.

Moreover, our findings demonstrate that within Senegal there appears to be a persisting gap between what providers are seeing in the field and how Senegalese governments and agencies have been able to respond to their observations and insights. Given that collaboration between providers, service agencies, and policy and regulatory officials is so crucial, in Senegal it may be useful to reinforce the WHO’s public health strategy for palliative care detailed by Stjernswärd and colleagues (2007). That approach involves a mutual, iterative process of integrating feedback that is top-down (from the government and other large organizations) as well as bottom-up (from providers and community health centers).

Participants specifically identified that financial support from the Senegalese government could come in the form of expanded education and training opportunities, which are areas that other countries have also identified as needing development (Drenth et al., 2017; Ntizimira et al., 2014; Rhee, Luyirika, et al., 2017). Senegal lacks a national curriculum for educating clinicians in palliative care (Rhee, Luyirika, et al., 2017), resulting in a piecemeal approach that puts the responsibility for advanced training on clinicians. Most clinicians in this study had pursued palliative care because of a personal interest in the kind of care offered by a palliative care philosophy. Three participants who lead palliative care services described how their personal experience of a loved one having a terminal illness, without access to palliative care, prompted them to introduce palliative care to their organization. Some professionals had formal palliative care training, while others had not, and all recognized the necessity of training both specialists and generalists in palliative care principles. Given that there are a limited number of training centers in Sub-Saharan Africa, Senegal could consider opening its own palliative care training center or encourage clinicians to use online training (Rawlinson et al., 2014). As previously mentioned, another factor critical to broadening access to palliative care is its integration into the national healthcare system, something not yet realized in Senegal (Gueye, 2016), despite its successful integration in other African countries such as Uganda and Rwanda (Fraser et al., 2017; Luyirika et al., 2022; Rhee, Garralda, et al., 2017).

Finally, the participants in our study noted that future efforts at expansion and integration of palliative care will be most successful if they consider the particular cultural context in the country (Afolabi et al., 2021). Palliative care originated in Western medical models, where a good death involves relieving suffering, acceptance, and avoiding unnecessary treatment (Cain & McCleskey, 2019). However, not all cultures and people share these views on death and dying. For example, in Taiwan, receiving palliative care is seen as a sign of giving up (Pie-Lin et al., 2019), and in countries like Croatia and Saudi Arabia, people avoid discussions of death and dying, especially around the patient, for fear of upsetting them (Kirn, 1998). In an African context, discussing death in front of a sick person is seen as taboo, and in the neighboring and majority Muslim country of Mauritania, the idea of having an incurable illness does not fit within the beliefs of Islam, and as such patients should not be told they have an incurable illness (Fearon et al., 2019). In this study, some participants reported that death and serious aspects of illness are not shared with the patient, to avoid exhausting and upsetting them, a practice also seen in other low- and middle-income countries (Hannon et al., 2016) and even as a barrier by clinicians in Nigeria and Cameroon (Tadipatri et al., 2021). Another contribution of this study is identifying similarly important attitudes and beliefs around death and dying that could be built into public education efforts to enhance the likelihood of dissemination and uptake of palliative care in Senegal.

Religion influences beliefs about the communication of health information, agency in disease progression, and ultimate control over death. Likewise, prayer can be an intervention, just like any medical procedure or medication. And religious leaders are often as trusted as healthcare providers, if not more. Indeed, one physician shared a story of her family member traveling to visit their religious leader to die rather than turning to a healthcare provider for care. In addition to working with religious leaders, participants also mentioned the widely accepted involvement of traditional healers, who are often the first point of contact for care in Senegal and other parts of Africa (Agom et al., 2021; Hannon et al., 2016; Harding & Higginson, 2005). These results suggest that cooperation between physicians, religious leaders, and traditional healers could benefit patients. Attention to religious beliefs, cultural practices, and social norms is essential to the successful utilization of palliative care and may, in fact, help secure its adoption in certain situations.

The findings from this study should be considered in light of several limitations. First, the small, and relatively homogeneous, sample limits the generalizability of our results. All participants lived and worked near Senegal’s capital, Dakar, and palliative care services in other parts of Senegal, particularly rural areas, are even more fragmented and sparse. The perspective of individuals in other parts of the country deserves attention. Additionally, because we did not collect data from all our participants on their educational background and religious affiliation, we cannot know how these participant characteristics may have shaped our results, given that religion plays an important role in everyday life in Senegal, and more education is often correlated with more access to healthcare (Hahn & Truman, 2015). Our study also included a relatively small number of caregivers, although their inclusion is a first, complementing similar data from other African countries (Fearon et al., 2019; Powell et al., 2014). Future studies might undertake systematic efforts to recruit more, and more diverse, types of caregivers for their important perspective. Including patients with serious illness, though obviously challenging, would also provide a unique window into how palliative care is experienced firsthand. Language barriers also presented some limitations, as all interviews were conducted in French, a second language for the researcher conducting interviews, and some interviews included Wolof, a dialect commonly spoken in Dakar but not by the interviewer. This might have led to some difficulties in translation, although efforts were made to triangulate findings with native speakers.

As pointed out by Harding et al. (2013), additional research focused on palliative care is a critical need. Further research efforts and global efforts to expand palliative care services in Senegal should continue to consider and integrate local perspectives, practices, and knowledge regarding serious illness care, as well as consider the moral complexity of past global health interventions. Future research in Senegal could consider prioritizing the recruitment of caregivers and patients to investigate attitudes, preferences, and service needs from the perspective of those who receive it (see, e.g., Powell et al., 2014). Palliative care consumers recruited for this research should reflect diversity in their personal characteristics, religious perspectives, geographical location, and disease features to ensure the most comprehensive perspective.

This study helps to illustrate the state of palliative care in Senegal and the opportunities for its expansion. Countries with more developed palliative care structures, such as Kenya, Uganda, and South Africa, can serve as models to help address barriers and align with the WHO’s public health strategy for palliative care (Drenth et al., 2017; Fraser et al., 2017; Stjernswärd et al., 2007). Listening closely to clinicians, patients, and caregivers can help inform international efforts to improve palliative care in Senegal and expand upon the grassroots work already being done by clinicians in the country.

## Supplementary Material

igad129_suppl_Supplementary_Material
