# Supplementary material for: “A Soft Death”: Perceptions and Attitudes Toward Palliative Care in Senegal
Source: Innov Aging. 2023 Nov 17;8(4):igad129. doi: 10.1093/geroni/igad129 (PMC10986751; doi:10.1093/geroni/igad129)
Supplement: igad129_suppl_Supplementary_Material [file igad129_suppl_supplementary_material.docx]

**Online Supplementary Material**

Section A. Interview Questions for Clinicians

Demographics/Background

- What’s your date of birth?
- What city and country are you from?
- How long have you been working for your organization?
- What is your job with the organization?
  - Why did you join your organization?
  - What do you do in your role?
- What kind of education do you have in palliative care?
- How did you get into the field of palliative care?

Other Questions

- Tell me about your experience of providing palliative care in the home (if applicable)? What services and care do you provide? When you’re in the home (if applicable), who do you work with (patients, family members, other healthcare providers)? (Can you further develop it; can you give examples?)
- In your experience, what do patients and family members know about palliative care? What kind of education is needed about palliative care?
- Tell me what you think is positive about providing palliative care in the home (if applicable)? How have you seen it help people? (Can you further develop it; can you give examples?)
- In your experience, what difficulties/obstacles have you seen in providing palliative care in the home (if applicable)? (Can you further develop it; can you give examples?)
- In your experience, how much do patients and their family members want to know about their illness? How much do you inform patients and their families of the illness during decision-making about treatments?
- What can/needs to be improved in palliative home care? (Can you give some suggestions on how?)
  - Specifically, how can palliative care in Senegal be improved?
- What is unique about the Senegalese culture or the healthcare system in Senegal that influences how palliative care is provided? i.e., religion
- Is there anything more you want to add before we finish?

Section B. Interview Questions for Caregivers

Demographics/Background

- What’s your birth date?
- What city and country are you from?
- What is/was your relation to the patient you are/were providing care to?
- How long have you been/were you a caregiver?
- What kind of illness does/did the patient have?

Other Questions

- Please tell me what you know about palliative care.
- What kind of experience have you had with palliative care? How did the palliative care providers help you?
- When a loved one is in their final stage of living, what are the most important things patients need?
- What symptoms are/were the most challenging to manage?
- And what treatments are/were most helpful?
- In the final stage of living, what is the role of family members, relatives, and friends?
- Who makes the decisions about the care for a family member at this stage of life?
- Are you religious? If so, what religion, and how does your faith play into care and decision-making at the end of someone’s life?
  - When do you decide to stop treatment?
- How did the patient’s illness and your caregiving affect you?
- Is there anything more you want to add before we finish?

Section C. Interview Questions for Public Health Professor

- What is the history of healthcare at the end of life in Senegal?
- Culturally, is there a preference for quantity or quality of life?
- When a person is sick, how much do they know about their illness? How much information do doctors tell them?
- At the end of life, who makes the medical decisions? For example, the individual or the family?
